# Supplementary material for: Viscoelastic optical nonlocality of low-loss epsilon-near-zero nanofilms
Source: Sci Rep. 2018 Jun 19;8:9335. doi: 10.1038/s41598-018-27655-z (PMC6008458; doi:10.1038/s41598-018-27655-z)
Supplement: Supplementary file 1 — Supplementary Information [file 41598_2018_27655_MOESM1_ESM.pdf]

Supplementary information for:

Viscoelastic optical nonlocality of low-loss epsilon-near-zero nanofilms

Domenico de Ceglia[1,*], Michael Scalora[2], Maria A. Vincenti[3], Salvatore Campione[4], Kyle Kelley[5], Evan L. Runnerstrom[5], Jon-Paul Maria[5], Gordon A. Keeler[4], and Ting S. Luk[4,6]

[1] *Department of Information Engineering, University of Padova, Italy*
[2] *US Army AMRDEC, Charles M. Bowden Research Laboratory, Redstone Arsenal (AL), USA*
[3] *Department of Information Engineering, University of Brescia, Italy*
[4] *Sandia National Laboratories, Albuquerque (NM), USA*
[5] *Department of Materials Science, North Carolina State University, Raleigh (NC), USA*
[6] *Center for Integrated Nanotechnologies (CINT), Sandia National Laboratories, Albuquerque (NM), USA*
[*] *Corresponding author:* domenico.deceglia@unipd.it

Supplementary Note 1: Derivation of the transport equations

Equations 1 and 2 in the main text are derived from the microscopic kinetic theory, as outlined in supplementary ref. 1. Here we summarize the main points of the derivation. We start from the Boltzmann equation, which provides the statistical description of the evolution of the electrons plasma in terms of electrons distribution function, as

$$\frac{\partial f}{\partial t} + (\boldsymbol{v}_m \cdot \nabla) f - \frac{e}{m}(\boldsymbol{E} + \boldsymbol{v}_m \times \boldsymbol{B}) \cdot \frac{\partial f}{\partial \boldsymbol{v}_m} = \left(\frac{\partial f}{\partial t}\right)_{coll} \tag{S1}$$

where $f(\boldsymbol{r}, \boldsymbol{v}_m, t)$ is the distribution function, $\boldsymbol{v}_m$ the microscopic electron velocity, m the electron mass, $-e(\boldsymbol{E}+\boldsymbol{v}_m \times \boldsymbol{B})$ the Lorentz force acting on the electrons, and $\left(\frac{\partial f}{\partial t}\right)_{coll}$ is an operator that accounts for collisions (with ions and between electrons). The next step is the conversion of the Boltzmann equation into a set of fluid-dynamic equations that describe the time evolution of macroscopic quantities, i.e., the velocity moments of the distribution function. These moments are measurable, macroscopic variables such as temperature, pressure, mean velocity, current density, and stress tensor. We consider the first three velocity moments. The first is the electron density, which is the zero-order velocity moment obtained by integrating the distribution function in the velocity space, $n(\boldsymbol{r}, t) = \int f(\boldsymbol{r}, \boldsymbol{v}_m, t)\, d\boldsymbol{v}_m$. The second is the first-order velocity moment divided by the electron density, i.e., the average (macroscopic) velocity, $\boldsymbol{v}(\boldsymbol{r}, t) = \frac{\int \boldsymbol{v}_m f(\boldsymbol{r}, \boldsymbol{v}_m, t) d\boldsymbol{v}_m}{\int f(\boldsymbol{r}, \boldsymbol{v}_m, t) d\boldsymbol{v}_m} = \frac{\int \boldsymbol{v}_m f(\boldsymbol{r}, \boldsymbol{v}_m, t) d\boldsymbol{v}_m}{n(\boldsymbol{r}, t)}$. The (macroscopic) current density is simply $\boldsymbol{J}(r, t) = -en(\boldsymbol{r}, t)\boldsymbol{v}(\boldsymbol{r}, t) = -e \int \boldsymbol{v}_m f(\boldsymbol{r}, \boldsymbol{v}_m, t)\, d\boldsymbol{v}_m$, and it is approximated to the first order $\boldsymbol{J}(r, t) = -en_0 \boldsymbol{v}(\boldsymbol{r}, t)$ in the main text. For higher-order moments, it is useful to transform the distribution function in the Lagrange comoving frame, i.e., $f^L(\boldsymbol{r}, \boldsymbol{v}_m, t) = f(\boldsymbol{r}, \boldsymbol{v}_m + \boldsymbol{v}, t)$. In this frame, the macroscopic velocity is zero and the second-order velocity moment, i.e., the pressure (stress) tensor, is written as $\boldsymbol{\Sigma}(\boldsymbol{r}, t) = m \int \boldsymbol{v}_m \otimes \boldsymbol{v}_m f^L(\boldsymbol{r}, \boldsymbol{v}_m, t)\, d\boldsymbol{v}_m$.

Next, the equations of motion for these macroscopic quantities (velocity moments) are retrieved by transforming the Boltzmann equation in an infinite chain of coupled equations for the moments of the distribution functions. The chain is truncated to the second order as follows.

The first equation (zero-order moment equation) is obtained by integrating the Boltzmann equation over the velocity space and assuming collisions with conservation of charge, mass and electron density (no recombination), hence $\int \left(\frac{\partial f}{\partial t}\right)_{coll} d\boldsymbol{v}_m = 0$. This leads to the usual continuity equation, $\frac{\partial n}{\partial t} + \nabla \cdot (n\boldsymbol{v}) = 0$. Eq. (3) in the main text is the linearization of this equation around the equilibrium, i.e., it is obtained by expanding the density as $n = n_0 + \delta n$. The first-order moment equation is obtained by multiplying the Boltzmann equation by $m\boldsymbol{v}_m$ and integrating in the velocity space. This yields the transport equation:

$$mn\frac{\partial v}{\partial t} + \nabla \cdot \boldsymbol{\Sigma} + mn(\boldsymbol{v} \cdot \nabla)\boldsymbol{v} - ne\boldsymbol{E} = \int m\boldsymbol{v}_m \left(\frac{\partial f}{\partial t}\right)_{coll} d\boldsymbol{v}_m \quad \text{(S2)}$$

In the relaxation time approximation, the first moment of the collision term assumes the simple expression $\int m\boldsymbol{v}_m \left(\frac{\partial f}{\partial t}\right)_{coll} d\boldsymbol{v}_m = -mn\gamma\boldsymbol{v}$, in which γ represents scattering of electrons with ions and impurities. Since $\boldsymbol{\Sigma}$ is symmetric, the isotropic part of the pressure tensor (i.e., the scalar pressure P) is separated from the viscous term $\boldsymbol{\Pi}$ as follows: $\boldsymbol{\Sigma} = P\boldsymbol{I} + \boldsymbol{\Pi}$, where $\boldsymbol{I}$ is the identity matrix, $P = \frac{1}{3}Tr(\boldsymbol{\Sigma})$ is the trace of $\boldsymbol{\Sigma}$ and $\boldsymbol{\Pi}$ is the traceless part of the stress tensor. Eq. (1) in the main text is the linearization of equation (S2) around the equilibrium, i.e., expanding density and pressure as $n = n_0 + \delta n$ and $P = P_0 + \delta P$. Transport effects due to the magnetic Lorentz force $-e\boldsymbol{v} \times \boldsymbol{B}$ and the convective term $mn(\boldsymbol{v} \cdot \nabla)\boldsymbol{v}$ play a central role in harmonic generation problems, but they can be neglected in our treatment in which only linear, nonlocal phenomena are investigated. Eq. (S2) generalizes classical hydrodynamic theories by introducing viscosity, therefore it describes electrons as a viscous fluid. It is important to remember that other classical approaches to describe the motion of conduction electrons can be derived from the Boltzmann equation by adopting different approximations. For example, the local Drude model treats conduction electrons in plasmonic materials as an incompressible gas, in which both elasticity and viscosity are neglected, i.e., $\boldsymbol{\Sigma} = \boldsymbol{0}$ in eq. (S2). The Bloch hydrodynamic theory, another classical and widely adopted model, considers only the isotropic part of the stress tensor, i.e., hence $\boldsymbol{\Pi} = \boldsymbol{0}$ and $\delta P \neq 0$.

Finally, the second-order moment equation (conservation of energy) is retrieved by multiplying the Boltzmann equation, eq. (S1), by $m\boldsymbol{v}_m \otimes \boldsymbol{v}_m$ and integrating over the velocity space:

$$\frac{\partial \Sigma}{\partial t} + (\boldsymbol{v} \cdot \nabla)\boldsymbol{\Sigma} + (\nabla \cdot \boldsymbol{v})\boldsymbol{\Sigma} + (\boldsymbol{\Sigma} \cdot \nabla) \otimes \boldsymbol{v} + \boldsymbol{v} \otimes (\boldsymbol{\Sigma} \cdot \nabla) + \nabla \cdot \Xi = \boldsymbol{X} \quad \text{(S3)}$$

where the second-order collision term is $\boldsymbol{X} = m\int \boldsymbol{v}_m \otimes \boldsymbol{v}_m \left(\frac{\partial f}{\partial t}\right)_{coll} d\boldsymbol{v}_m$. The third-order moment $\Xi(\boldsymbol{r}, t) = m\int \boldsymbol{v}_m \otimes \boldsymbol{v}_m \otimes \boldsymbol{v}_m f^L(\boldsymbol{r}, \boldsymbol{v}_m, t)\, d\boldsymbol{v}_m$ is neglected due to the second-order

truncation in our treatment, an approximation that is justified for small values of the parameter $\alpha = \frac{v_F}{L \max\{\omega, \gamma\}}$ [see the main manuscript after eq. (1) for parameter definitions].

The second-moment equation is then decomposed into an isotropic part and a traceless part by substituting $\mathbf{\Sigma} = P\boldsymbol{I} + \mathbf{\Pi}$ in eq. (S3). For the second moment of the collision term, we use the same approach of supplementary ref. 1, in which the Krook expression $\left(\frac{\partial f}{\partial t}\right)_{coll} = -\gamma_{VE}(f - f_0)$ is adopted. For a Fermi gas, the equilibrium distribution function is equal to the Fermi function, $f_0 = f_F$, and the right-hand side of eq. (S3) becomes $m \int \boldsymbol{v}_m \otimes \boldsymbol{v}_m \left(\frac{\partial f}{\partial t}\right)_{coll} d\boldsymbol{v}_m = -\gamma_{VE}(\mathbf{\Sigma} - \boldsymbol{I}P_F)$. Since the Fermi pressure $P_F = P$, the collision moment is simply $-\gamma_{VE}\mathbf{\Pi}$, i.e., it only acts on the traceless part of the stress tensor.

The trace of eq. (S3) reads as

$$\frac{\partial P}{\partial t} + (\boldsymbol{v} \cdot \nabla)P + \frac{5}{3}P(\nabla \cdot \boldsymbol{v}) + \frac{2}{3}(\mathbf{\Pi} \cdot \nabla) \cdot \boldsymbol{v} = 0 \tag{S4}$$

while the traceless, remaining part of eq. (S3) is

$$\frac{\partial \Pi}{\partial t} + (\boldsymbol{v} \cdot \nabla)\mathbf{\Pi} + (\nabla \cdot \boldsymbol{v})\mathbf{\Pi} + (\mathbf{\Pi} \cdot \nabla) \otimes \boldsymbol{v} + \boldsymbol{v} \otimes (\mathbf{\Pi} \cdot \nabla) - \frac{2}{3}\boldsymbol{I}(\mathbf{\Pi} \cdot \nabla) \cdot \boldsymbol{v} + P\left(\nabla \otimes \boldsymbol{v} + \boldsymbol{v} \otimes \nabla - \frac{2}{3}\boldsymbol{I}\nabla \cdot \boldsymbol{v}\right) = -\gamma\mathbf{\Pi} \tag{S5}$$

Considering for the kinetic pressure an expression equal to that of a degenerate Fermi gas at $T = 0$ K, $P = \frac{\hbar^2}{5m^*}(3\pi^2)^{2/3} n^{5/3}$, the term $\frac{2}{3}(\mathbf{\Pi} \cdot \nabla) \cdot \boldsymbol{v}$ vanishes in eq. (S4). This means that the linearized version of eq. (S4) is equal to eq. 2(a) of the main text.

Terms that depend on the product of $\mathbf{\Pi}$ by $\boldsymbol{v}$ in eq. (S5) vanish in our perturbative, linear approximation, therefore eq. (S5) reduces to eq. 2(b) of the main text.

Supplementary Note 2: Additional observations of higher-order modes

The trend of higher-order resonances has been consistently observed in films with thickness smaller than 25 nm. In Fig. S1 we show the ellipsometric quantity $\Psi = \tan^{-1}|R_p|/|R_s|$ for three additional samples having different thicknesses of 16.2 nm, 21.3 nm, and 38.8 nm. The theoretical fit obtained with the nonlocal viscoelastic model is reported together with the measured data. The trend is in agreement with Fig. 2 of the main manuscript: the larger-thickness film of 38.8 nm shows little nonlocal behavior (blue curve), hence it displays no higher-order resonances, while the two thinner films show one or two additional higher-order resonances (green and red curves).

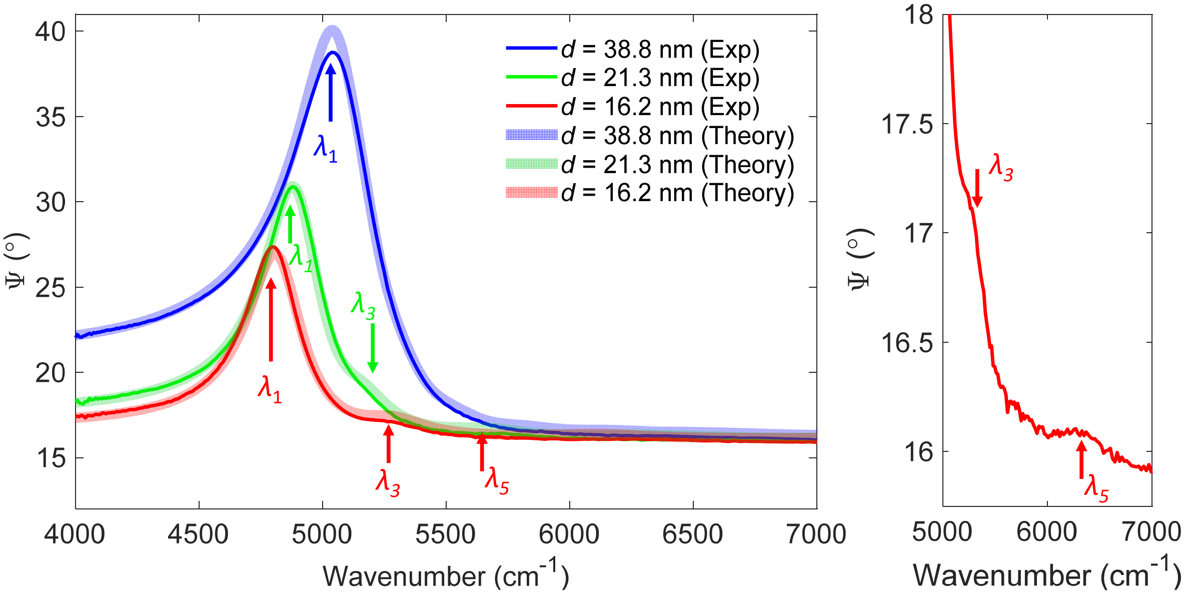

Fig. S1. On the left, measured and calculated (via nonlocal theory) spectra of the ellipsometric parameter Ψ (reported in degrees) for three samples. On the right, zoom of the measured spectrum for the d = 16.2 nm to highlight the resonances at λ_3 and λ_5. The properties of the three samples are summarized in table S1.

We stress that these samples were not prepared for ATR, therefore the spectra were taken from the air side and the modes appear as shallower resonances (see also Fig. 1 in the manuscript). The enlarged view of the thinnest-film spectrum clearly shows the two additional resonances (right panel of Fig. S1). As for the samples presented in the manuscript, film thicknesses were measured with X-ray reflectivity (XRR), while carrier concentrations and mobilities are determined with Hall-effect measurements. The film properties are summarized in Table S1.

	Thickness d (nm)	Carrier conc. n_0 (cm^{-3})	Mobility (cm^2/Vs)
**Film 1**	38.8	3×10^{20}	310
**Film 2**	21.3	2.7×10^{20}	290
**Film 3**	16.2	2.53×10^{20}	280

Table S1. Measured parameters of the three additional films with differing thicknesses.

Supplementary references

1. Tokatly, I. and Pankratov, O. Hydrodynamic theory of an electron gas. *Phys. Rev. B* **60**, 15550-15553 (1999).
